# Design and Synthesis of Analogues of Marine Natural Product Galaxamide, an *N*-methylated Cyclic Pentapeptide, as Potential Anti-Tumor Agent in Vitro

**DOI:** 10.3390/md14090161

**Published:** 2016-09-03

**Authors:** Jignesh Lunagariya, Shenghui Zhong, Jianwei Chen, Defa Bai, Poonam Bhadja, Weili Long, Xiaojian Liao, Xiaoli Tang, Shihai Xu

**Affiliations:** 1 Department of Chemistry, Life Science School, Jinan University, Guangzhou 510632, China; jignesh.lunagariya@gmail.com (J.L.); shenghuizhong@foxmail.com (S.Z.); 18826237580@163.com (J.C.); gallopfeel@gmail.com (W.L.); 2 College of Pharmacy, Jinan University, Guangzhou 510632, China; tbaidf@jnu.edu.cn; 3 Institute of Biomineralization and Lithiasis Research, Department of Chemistry, Life Science School, Jinan University, Guangzhou 510632, China; poonambhadja@gmail.com; 4 Alpert Medical School, Brown University, 55 Claverick St. 4th Floor, Providence, RI 02903, USA

**Keywords:** anti-tumor, apoptosis, cyclic pentapeptide, galaxamide analogues, macrocyclisation

## Abstract

Herein, we report design and synthesis of novel 26 galaxamide analogues with *N*-methylated cyclo-pentapeptide, and their in vitro anti-tumor activity towards the panel of human tumor cell line, such as, A549, A549/DPP, HepG2 and SMMC-7721 using MTT assay. We have also investigated the effect of galaxamide and its representative analogues on growth, cell-cycle phases, and induction of apoptosis in SMMC-7721 cells in vitro. Reckon with the significance of conformational space and *N*-Me aminoacid (aa) comprising this compound template, we designed the analogues with modification in *N*-Me-aa position, change in aa configuration from l to d aa and substitute one Leu-aa to d/l Phe-aa residue with respective to the parent structure. The efficient solid phase parallel synthesis approach is employed for the linear pentapeptide residue containing *N*-Me aa, followed by solution phase macrocyclisation to afford target cyclo pentapeptide compounds. In the present study, all galaxamide analogues exhibited growth inhibition in A549, A549/DPP, SMMC-7721 and HepG2 cell lines. Compounds **6**, **18**, and **22** exhibited interesting activities towards all cell line tested, while Compounds **1**, **4**, **15**, and **22** showed strong activity towards SMMC-7221 cell line in the range of 1–2 μg/mL IC_50_. Flow cytometry experiment revealed that galaxamide analogues namely Compounds **6**, **18**, and **22** induced concentration dependent SMMC-7721 cell apoptosis after 48 h. These compounds induced G0/G1 phase cell-cycle arrest and morphological changes indicating induction of apoptosis. Thus, findings of our study suggest that the galaxamide and its analogues 6, 18 and 22 exerted growth inhibitory effect on SMMC-7721 cells by arresting the cell cycle in the G0/G1 phase and inducing apoptosis. Compound **1** showed promising anti-tumor activity towards SMMC-7721 cancer cell line, which is 9 and 10 fold higher than galaxamide and reference DPP (cisplatin), respectively.

## 1. Introduction

The marine environment is the vital source of living organisms [1,2], which provides diversity of natural products that pave the way for medicinal scientists to discover well-suited bioactive compounds for the biological systems [3]. Among them, peptides are very interesting target following their important role as hormones [4], neurotransmitters [5], growth factors [6], ion channel ligands [7], or anti-infectives [8]. Nearly, more than 7000 naturally occurring peptides have been identified [9], including several bioactive peptides. Peptides and their homologous compounds (proteins and antibodies) can be used in multiple pathologies, including allergy and asthma [10], arthritis [11], cancer [12], cardiovascular diseases [13], diabetes [14], gastrointestinal dysfunction [15], HIV [16], infective diseases [17], inflammation [18], pain [19], and so on.

At present, around 100 peptide medicines on the market, and which is expected to grow considerably [20], with about 140 currently in clinical trials and more than 500 therapeutic peptides in preclinical development [9].

In recent time, approve drug and candidates from small molecules in clinical trials are decreasing, which led scientists to pay more attention towards peptides and other alternatives [21]. The probability of regulatory approval is 20% for the peptide drugs with respect to 10% for the small molecule [22,23], as the small molecules are not selective and can accumulate in specific organs such as the kidney and liver, resulting in severe toxic side effects [24].

**Figure 1:**
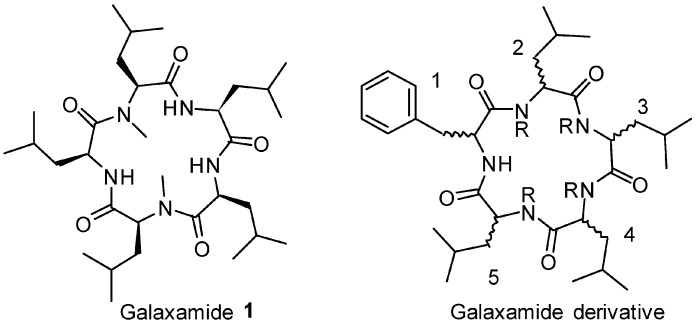
Structure of galaxamide and general structure of its derivative.

In peptide drug portfolio, around half of the peptides in clinical trial have target indication in oncology, metabolic, cardiovascular and infectious diseases [24,25]. In particular, cyclic pentapeptides have been demonstrated as anti-tumor agent, and shown cytotoxicity towards several types of tumors, including pancreatic [26,27,28], colon [29,30,31,32], breast, prostate, and melanoma cancers [26,33,34], which proved its high potential in targeting the cancer. Following discovery of natural product sansalvamide, a cyclic depsipeptide, several reports have been demonstrated anti-tumor activity of Sansalvamide A analogues, including *N*-methylsanslavamide A analogues as a variety of anti-tumor agent by Silverman group [26]. Accordingly, McAlpine group also published numerous articles on San A, such as, analogues as treatment of MSS colon cancer [30], synthesis of analogues as potential anti-tumor [34], and potent derivative against pancreatic cancer cell lines [27]. Along the same line, galaxamide **1**, an *N*-methylated cyclic pentapeptide, has been discovered from marine algae *Galaxaura filamentosa* with structural determination and first total synthesis by our group [35]. Galaxamide 1and general structure of its analogues have shown in Figure 1. Galaxamide and its analogues have also shown potential anti tumor activity [36]. All of these analogues, including sansalvamide analogues, have clearly suggested that the incorporation of *N*-Me and d aa alter the conformational space and hydrogen bonding, which consequently play a crucial role in the cytotoxicity and other pharmacokinetic property of the compound. Hence, in continuation of our interest to find more potent lead compound as anti-tumor agent on the basis of conformational space and hydrogen bonding, we report the solid phase synthesis of 26 galaxamide analogues with modification in *N*-Me-aa position, change in aa configuration from l to d aa and substitute one Leu-aa to d/l Phe-aa residue with respective to the parent structure, and their in vitro anti-tumor activity against A549, A549/DPP, SMMC-7721 and HepG2 cell lines using MTT assay. We further investigated its mechanism of action by detecting cell morphology, cell-cycle progression, and apoptosis using SMMC-7721 as representative cell line model.

## 2. Results

### 2.1. Chemistry

Recently, in most cases, synthesis of cyclo pentapeptide was employed on segment based solution phase synthetic strategy [27,30,34,35,36]. Synthesis of galaxamide and its analogues have been reported by our group with a similar strategy [35,36].

**Scheme 1:**
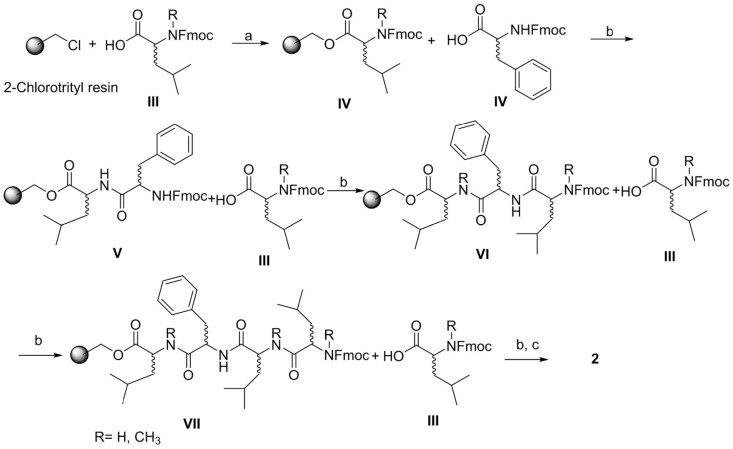
Reaction scheme for linear peptide on solid phase. Reagents and conditions: a, (i) dichloro methane (DCM), *N*,*N*-Diisopropylethylamine (DIPEA) 4 eq., room temperature (rt); (ii) DCM/MeOH/DIPEA (4/5/1), 30 min, rt; b, (i) 20% piperidine in *N*,*N*-Dimethylformamide (DMF) (2 × 10 min); (ii) DIPEA 2 eq., DEPBT 2 eq., DCM/DMF (1/1), 3 h, rt; c, 1% TFA in DCM for (2 × 30 min), rt.

**Scheme 2:**
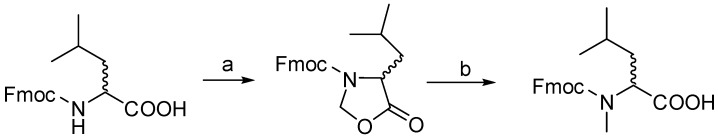
Freidinger synthesis of *N*-methylated Leucine. Reagents and conditions: a, (CH_2_O)*n*, PTSA (cat.), toluene, azeotropic removal of water; b, Et_3_SiH, CF_3_COOH, CHCl_3_.

In present study, we synthesized linear pentapeptide using Fmoc peptide synthetic strategy with 2-chloro trityl resin solid phase (Scheme 1), which is very proficient in a way to efficient, speed, easy to handle and high purity of crude product. Synthesis of d/l *N*-Me Leu aa residue were performed as per reported method by Zhang et al. [37] (Scheme 2). Briefly, *N*-Fmoc protected aa was converted into **5-**oxazolidinones derivative using para formaldehyde in the presence of catalytic amount of *p*-toluenesulfonic acid (PTSA) in toluene under azeotropic water removal condition, followed by reduction with triethylsilane in the presence of lewis acid to provide *N*-Me aa. This method is more convenient over *N*-Me with sodium hydride/methyl iodide according to Benoiton’s protocol [38].

First aa residue was immobilized on resin using 4.0 eq. *N*,*N*-Diisopropylethylamine (DIPEA) in dichloro methane (DCM) for 2 h at room temperature (rt). Subsequent aa coupling including *N*-me aa was performed via 3-(Diethoxyphosphoryloxy)-1,2,3-benzotriazin-4(3H)-one (DEPBT) mediated activation in DCM with DIPEA as base. DEPBT was chosen as more convenient for *N*-Me aa coupling and negligible racemization [39]. Solid-phase reactions were monitored by use of a qualitative Kaiser test [40] for the detection of primary amines and the chloranil test [41] for detection of secondary amines. Coupling reaction was completed within 2 h except for coupling with *N*-Me aa, which generally took 8–10 h. Upon cleavage from solid support with 1% TFA in DCM linear pentapeptide was obtained in 50%–60% overall yield with 90%–95% purity which was verified by RP-HPLC.

**Scheme 3:**
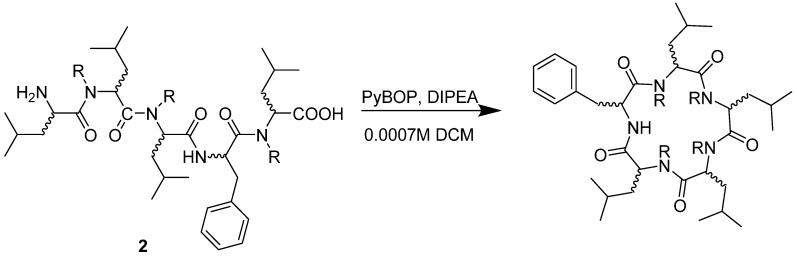
Macrocylclisation of linear pentapeptide.

The linear pentapeptide was isolated by precipitation from diethylether, collected by centrifugation, which can be used for macrocyclisation without further purification. Key challenge for macrocyclisation is lower yield due to many intermolecular reactions, resulting into various complex oligomeric products. Recently, some advancement on macrocyclisation reaction of pentapeptide have been suggested that (Benzotriazol-1-yloxy)tripyrrolidinophosphonium hexafluorophosphate (PyBOP) is better coupling reagent for obtaining higher yield with short reaction time [42]. After screening range of dilution of solvent DCM and base equivalent, employing PyBOP as a coupling reagent for macrocyclisation, promising reaction condition with 0.0007 M dilution of solvent DCM in the presence of 2 eq. of base DIPEA and 2 eq. PyBOP have been established (Scheme 3). After 24 h reaction, targeted cyclo pentapeptides were obtained in 44%–66% yield after preparative RP-HPLC purification.

#### Structure of Macrocyclic Analogues

**Figure 2:**
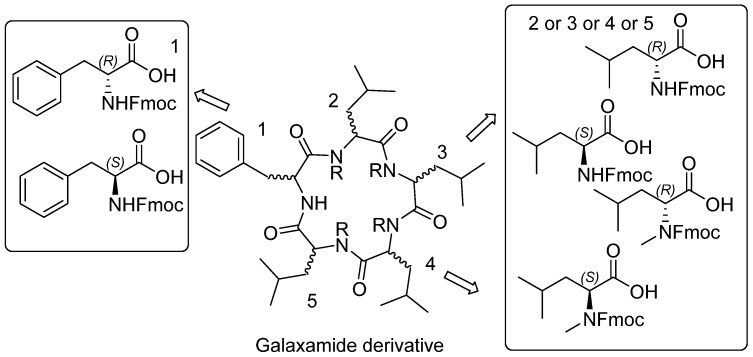
Amino acid used at various position for galaxamide derivative.

We designed and synthesized three subsets of galaxamide derivatives. These were mainly differentiated through *N*-Me position, addition to that multi changes have been incorporated with position and number of d configuration aa residues, which was common in all three subset (Figure 2).

**Figure 3:**
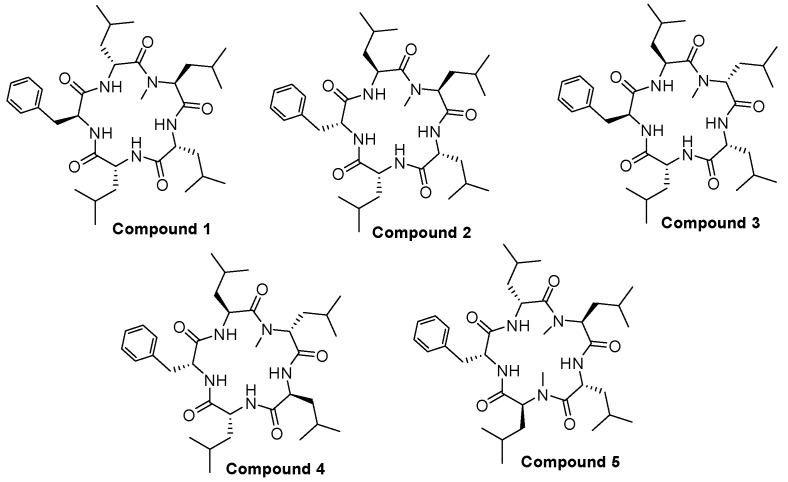
*Subset 1*: *N*-Me at third position galaxamide derivative.

**Figure 4:**
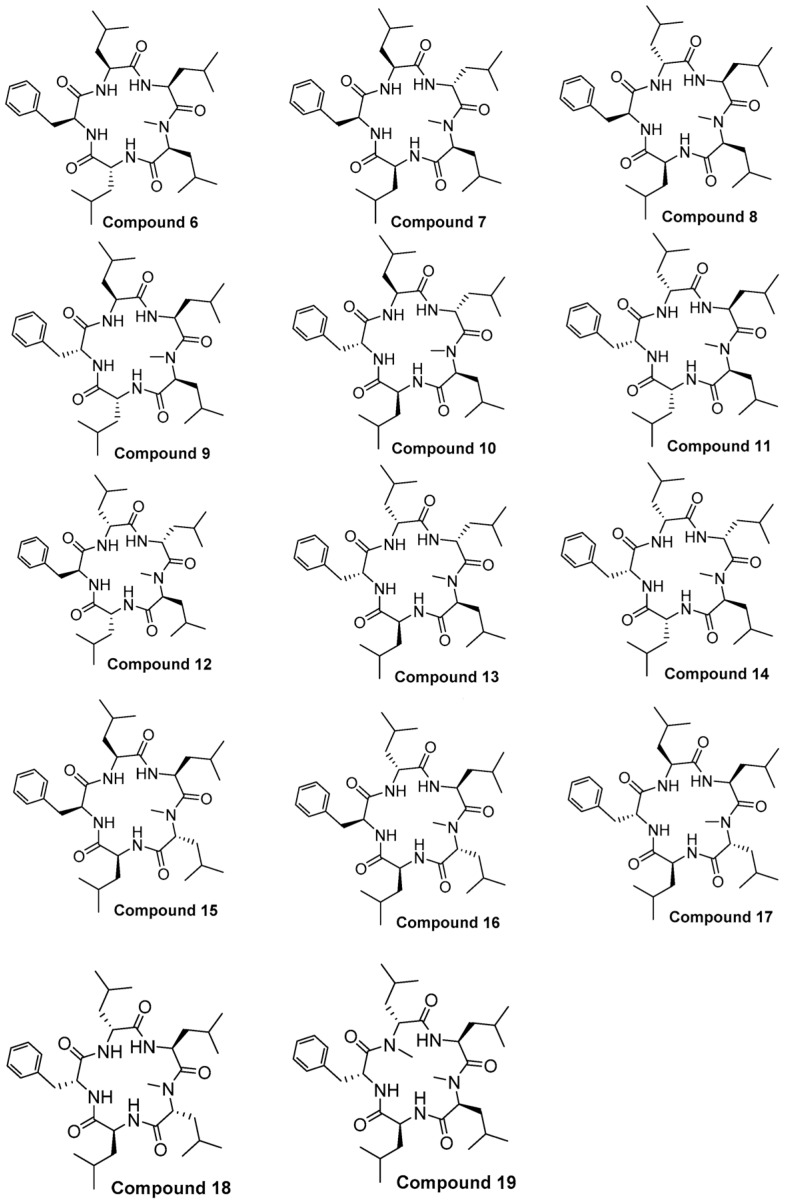
*Subset 2*: *N*-Me at fourth position galaxamide derivative.

**Figure 5:**
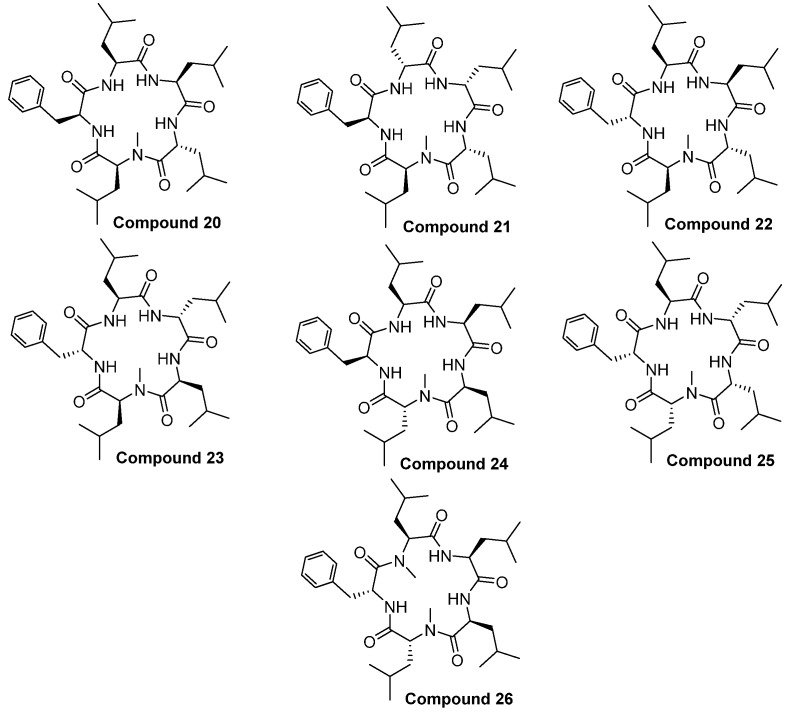
*Subset 3*: *N*-Me at fourth position galaxamide derivative.

Some compounds also include two *N*-Me aa residue product (Compound **5**, **19**, and **26**). The first subset of compounds contains *N*-Me aa residue at third position (Compound **1**–**5**, Figure 3). The second subset contain *N*-Me aa residue at fourth position (Compounds **6**–**19**, Figure 4). The third subset contain *N*-Me aa residue at fifth position (Compounds **20**–**26**, Figure 5).

### 2.2. Biological Activity

#### 2.2.1. Inhibitory Activity of Galaxamide and Its Analogues

**Figure 6:**
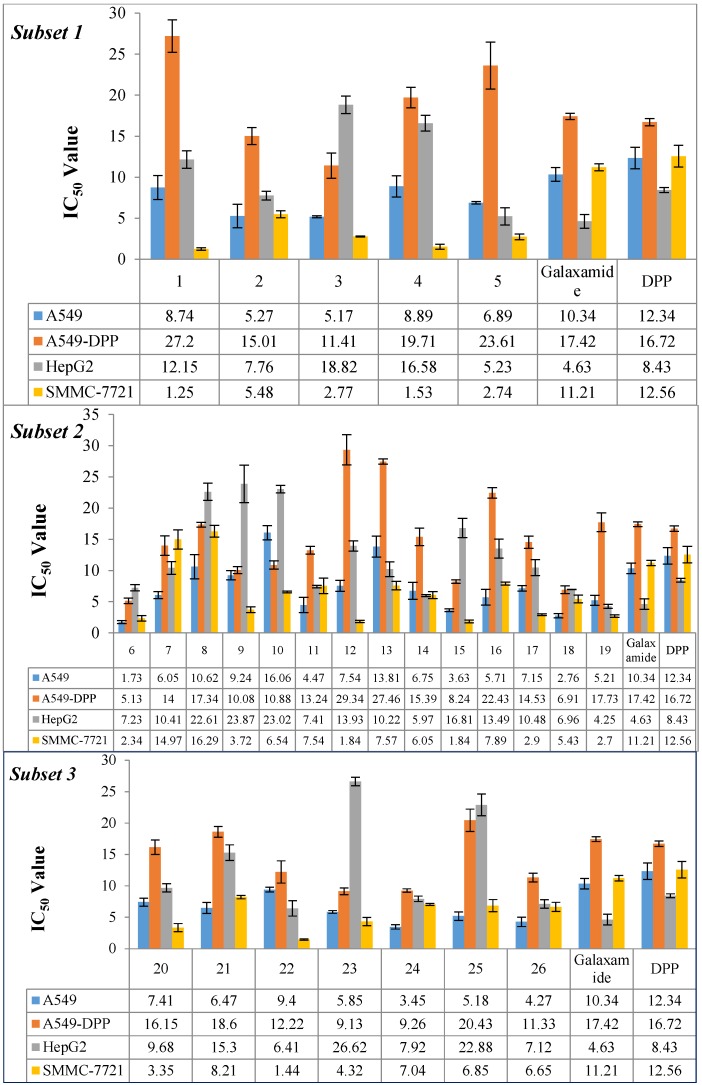
Cytotoxicity of compounds galaxamide and its three subset analogues against A549, A549/DPP, HepG2 and SMMC-7721 (IC_50_, μg/mL) using MTT assay. The IC_50_ values are reported as the means ± SD from three independent experiments. Lung cancer cell line A549, drug-resistant lung cancer cell line A549/DPP, human hepatocellular carcinoma HepG2 and SMMC-7721.

Galaxamide and its analogues were evaluated for their cytotoxicity against panel of tumor cell lines, such as, Lung cancer cell line A549, drug-resistant lung cancer cell line A549/DPP, human hepatocellular carcinoma HepG2, SMMC-7721 and also against human normal liver cell line L02 using MTT assay. These compounds selectively differently interact with cancer cell over normal liver cell line and exhibited less cytotoxicity to normal cell with >40 μg/mL IC_50_ value, except for the Compounds **14** and **15** which showed 34.63 and 36.87 μg/mL IC_50_ value and was also seven fold higher than galaxamide against HepG2. Compounds **6**, **18** and **22** exhibited interesting activities towards all cell line tested, while Compounds **1**, **4**, **15** and **22** showed strong activity towards SMMC-7221 cell line in the range of 1–2 μg/mL IC_50_. Additionally, we have further studied three Compounds **6**, **18** and **22** as representative of all these analogues to understand the pathway. We have chosen these compounds as they have expressed interesting activities towards all cell line and had moderate differences with Compounds **1**, **4** and **15** which showed strongest activity in SMMC-7721 cell line. The results are summarized in Figure 6.

#### 2.2.2. Galaxamide and Its Analogues Caused G0/G1 Arrest

**Figure 7:**
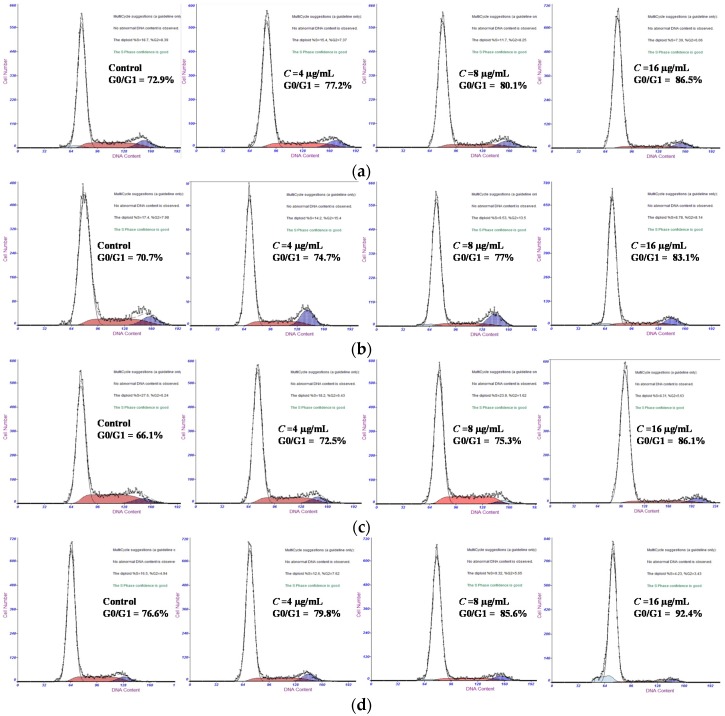
Galaxamide and its analogues caused G0/G1 phase arrest in SMMC-7721 cells. SMMC-7721 cells were incubated with or without 4, 8 and 16 μg/mL galaxamide (**a**) and compounds 6 (**b**), 18 (**c**) and 22 (**d**) for 48 h. Cell cycle was determined by PI staining using flow cytometry.

The cell cycle of cancer cells was assessed to continue the investigation into the toxicity of galaxamide and its analogues. Figure 7 illustrates the representative experimental results of propidium iodide (PI) fluorescence intensity, which was proportional to the DNA content and indicated the cell cycle phases. To investigate the mechanism of galaxamide and its analogues on cell growth inhibition, SMMC-7721 cells were exposed to representative compounds with the concentrations of 4, 8 and 16 μg/mL for 48 h before submitted to cell-cycle analysis. Result of this experiment showed that treatment of SMMC-7721 cells with these compounds promoted a considerable increase in the percentage of cells in the G0/G1 phase, which suggested that compounds caused cell-cycle arrest at G0/G1 phase in which the cell grows and prepares to synthesize DNA. Thus, these analogues compounds directly interact with the nucleus of cancer cells, and did not induce DNA fragmentation in cells, thereby altered the cell-cycle progression and inhibited growth of cancer cells.

#### 2.2.3. Galaxamide and Its Analogues Induced Apoptosis of SMMC-7721 Cells

**Figure 8:**
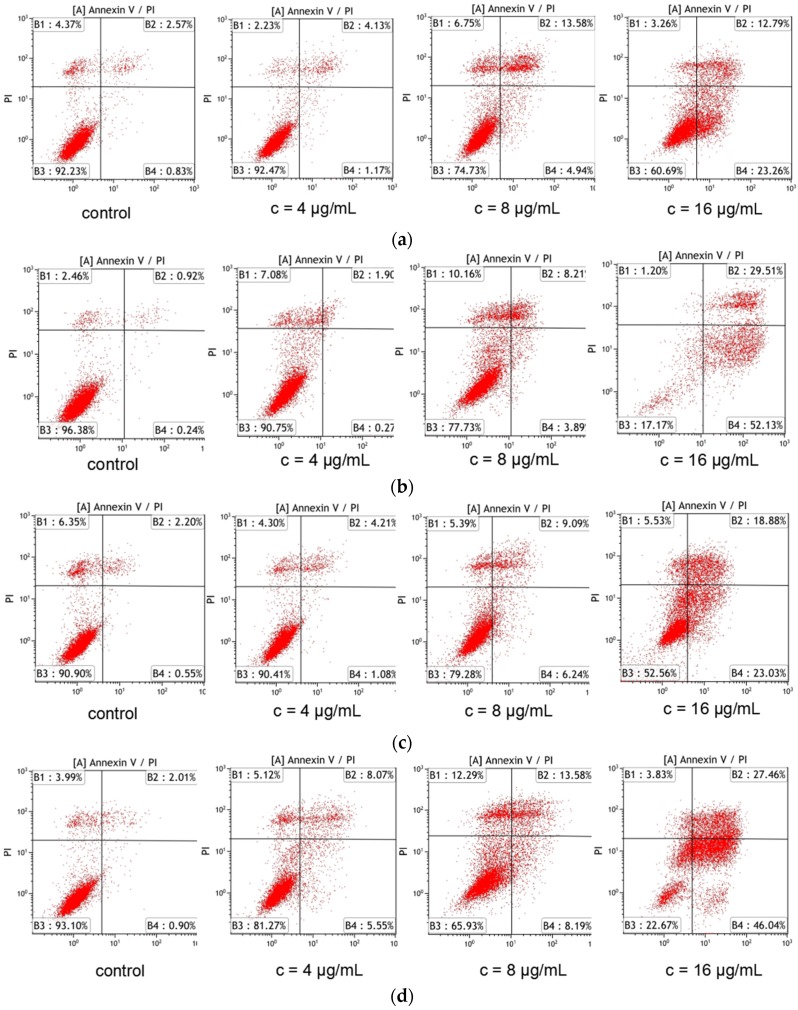
Galaxamide and its analogues induced apoptosis in SMMC-7721 cells. SMMC-7721 cells were incubated with or without 4, 8 and 16 μg/mL galaxamide (**a**) and compounds 6 (**b**), 18 (**c**) and 22 (**d**) for 48 h. The apoptosis of SMMC-7721 cells was determined by annexin V/PI staining. **B1** (the upper left) quadrant represents necrotic cells, **B2** (the upper right) quadrant represents late apoptotic and dead cells, **B3** (the bottom left) represents normal cells, **B4** (the bottom right) quadrant represents early apoptotic cells.

**Figure 9:**
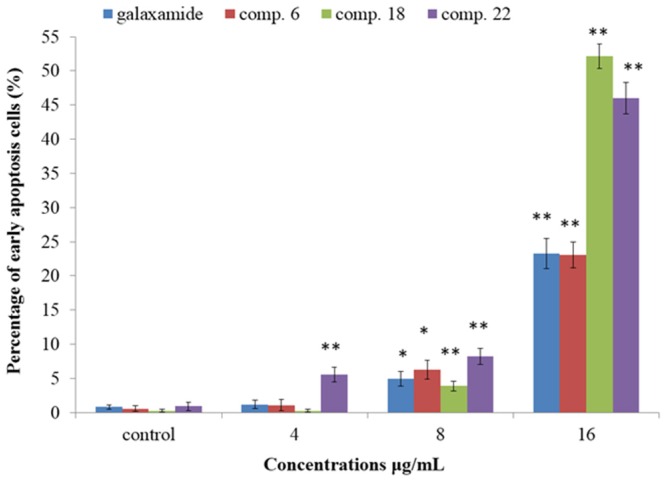
Galaxamide and its analogues induced apoptosis in SMMC-7721 cells. Annexin V positive cells of three independent experiments were shown in column statistics. Data expressed as mean ± SD. * *p* < 0.05; ** *p* < 0.01 vs. control

SMMC-7721 cell apoptosis was examined by flow cytometric analysis using annexin V/PI double staining after 48 h treatment of galaxamide and its analogues with different concentrations (4, 8 and 16 μg/mL). Apoptosis plays an important role in the treatment of cancer, as it is a popular target of cancer treatment strategies. The abundance of literature suggests that targeting apoptosis in cancer is feasible. As shown in Figure 8, the lower right quadrants accounted the early apoptotic rate, and column statistics of apoptotic rate presented in Figure 9. We found that the representative Compounds **6**, **18** and **22** induced SMMC-7721 cell apoptosis in dose-dependent manner. Hence, these analogues caused apoptosis in cancer cells through growth arrest during cell-cycle progression pathway.

#### 2.2.4. Morphological Changes of SMMC-7721 Cells Induced by the Treatment of Galaxamide and Its Analogues

**Figure 10:**
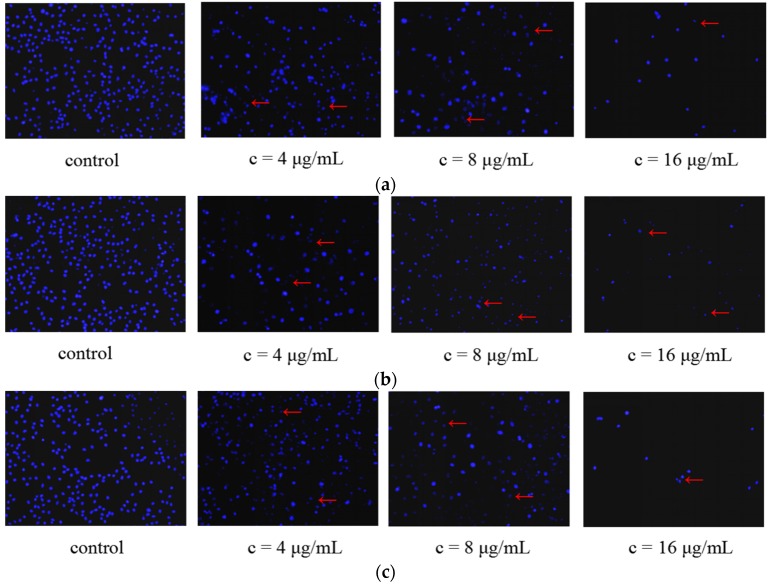

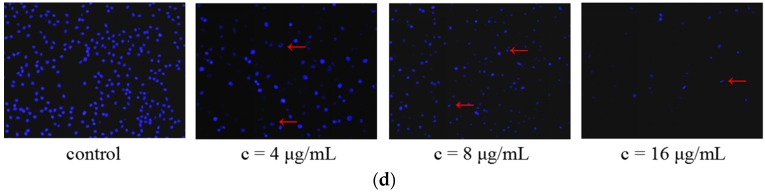
Morphological observation of galaxamide and its analogues-induced apoptosis in SMMC-7721 cells as indicated by Hoechst33342 staining. SMMC-7721 cells were incubated with or without 4, 8, 16 μg/mL galaxamide (**a**) and compounds 6 (**b**); 18 (**c**) and 22 (**d**) for 48 h, and then cell morphology was determined by Hoechst33342 staining with to visualize the nucleus (blue) under a fluorescent microscope at a magnification of 200×; scale bar, 50 μm. Arrows indicate apoptotic features (condensed chromatin and nuclear fragmentation).

Hoechst33342 staining was performed for the morphological alteration analysis of SMMC-7721 cells using fluorescence microscopy. As shown in Figure 10, treatment of SMMC-7721 cells with 4, 8 and 16 μg/mL galaxamide and representative Compounds **6**, **18**, **22** analogues resulted in cell detachment from cell culture plates. This action shows dose-dependent toxicity of evaluated compounds towards cancer cells. Herein, control cells exhibited normal morphology. However, galaxamide and its analogues-treated cells lost substrate attachment, became rounded, shrank, condensed their chromatin and displayed membrane blebbing. These morphological features shows that these compounds share cell growth inhibition by inducing apoptosis and cell growth arrest, eventually lead to cell death of cancerous cells.

## 3. Discussion

### 3.1. Structure-Activity Relationship

In our previous article, we have reported in vitro anti-tumor activity of few analogues of galaxamide with inclusion of d Phe and variation in number of d Leu aa residue, from which, one analogues showed promising anti-tumor activity against HepG2 with the three fold enhancement than galaxamide [36]. In continuation to find more potent compound, we have demonstrated 26 galaxamide analogues with inclusion of one d/l Phe aa residue, only one *N*-Me Leu residue at three, four and five position of macrocycle called *subset 1, subset 2* and *subset 3* (except Compounds **5**, **19** and **26** where two *N*-Me were introduced), respectively including variation in position and number of d Leu aa residue. In general, particular trend was unobserved in the structure-activity relationship as many investigations have recently suggested that the major factor between potency and structure involves conformational space which lock the macrocycle into its low energy conformation and thus binding to its biological target in that position with appropriately present side chain [27]. Furthermore, compounds mostly showed dissimilar trend in activity against all cancer cell line except A549 and A549/DPP where similar trend was observed in increase and decrease of cytotoxicity. All the compounds showed less activity compared to galaxamide in HepG2 cancer cell line, though showed comparable activity with DPP. This might be reason that two *N*-Me aa is favorable for HepG2 cancer cell as the Compounds **5**, **19**, and **26** with two *N*-Me showed comparable activity with galaxamide. Compound **6** which contain *N*-me at 4 and d Leu at 5 position showed strongest activity (1.73 and 5.73 μg/mL IC_50_) against A549 and A549/DPP, respectively followed by Compound **18**, which include d Leu at 2 and d *N*-Me Leu at 4 position showed excellent activity (2.76 and 6.91 μg/mL IC_50_, respectively). Compounds **1**, **4**, **12**, **15**, and **22** showed strongest activity in the range of 1–2 μg/mL IC_50_ value followed by Compounds **3**, **5**, **6**, **17**, and **19** which showed activity in the range of 2–3 μg/mL IC_50_ value against SMMC-7221 cell line. There was no particular structure-activity relationship (SAR) observed, though all *subset 1* compounds with *N*-Me at 3 position showed excellent activity. While strongest activity shown by Compound **1** from *Subset 1* had 1.25 μg/mL IC_50_ value which is 9 and 10 fold higher than parent galaxamide and current marketed drug DPP, respectively. In particular, from the SAR it is observed that the *N*-Me at 3 position is very critical which might change the conformation that boost the activity with enhancement of binding to the biological target.

### 3.2. Mechanism of Apoptosis Pathway

It is well known that apoptosis is a programmed process influence the density of a cell in multicellular organisms in order to regulate tissue homeostasis. It becomes a matter of life and death and the survival of the organism lies in the balance. Deregulation of cell proliferation and/or apoptosis leads to pathological conditions of cancer. The life spans of both normal and cancer cells within a living system are regarded to be substantially affected by the rate of apoptosis. The understanding of apoptosis events and its pathways has provided basic novel targets in the management and therapy for cancer. Two major pathways are involved in mammalian cells apoptosis—mitochondria-mediated intrinsic pathway and death receptor-mediated extrinsic pathway [43]. Thus, the chemopreventive agents that can modulate apoptosis, are often useful in cancer therapy. Recently, the anti-tumor activity of cyclic peptides has attracted much attention [44]. Many natural and synthetic cyclopeptides have been proven to be clinically effective for treating cancer could inhibit tumors by triggering cancer cell apoptosis. RA-V (deoxybouvardin), an unique natural cyclopeptide which was derived from the medical plant *Rubia yunnanensis*, can induce mitochondria-mediated apoptosis, exerted anti-tumor activity against human breast cancer [45]. Sansalvamide A, isolated from a marine fungus of the genus Fusarium, is a cyclic tetrapeptide exhibited better anti-tumor activity [33]. In this study, we have shown that galaxamide and its analogues resulted in an induction of apoptosis in dose-dependent manner in SMMC-7721 cells, confirmed by annexin V/PI staining assay. Therefore, inducing apoptosis might be the primary mechanism for the anti-cancer activity of galaxamide analogues. Moreover, higher concentration of representative galaxamide derivatives induced better apoptotic effect than galaxamide.

Cell cycle and apoptosis are considered to be two major regulatory mechanisms for cell growth. When specific checkpoints during the cell cycle are arrested, apoptotic cell death occurs [46]. These checkpoints maintain the fidelity of DNA replication, repair, and division. Moreover, most cancer therapeutics cause cell cycle arrest, and have been confirmed to be valuable agent for cancer treatment [47]. It has been reported that a cyclic-peptide sansalvamide analogue inhibits pancreatic cancer cell growth through G0/G1 cell-cycle arrest and apoptosis [28]. Herein, flow cytometric examination showed that the growth inhibition of SMMC-7721 cells induced by galaxamide and its analogues occurs through the cell cycle arrest in G0/G1 phase. Therefore, suggests that these agents may slow down the growth of cancer cells by artificially imposing the cell cycle checkpoint. G0/G1 phase arrest of cell cycle regulation provides an opportunity for cells to follow the apoptotic pathway. The G1 to S cell cycle progression is controlled by several cyclin-dependent kinase (CDK) complexes, the activities of which are dependent on the balance of cyclins and cyclin-dependent kinase inhibitors (CKIs). p53, the most extensively studied tumor suppressor, mediates a variety of anti-proliferative processes through cell cycle checkpoints, DNA repair and apoptosis [48]. Previous reports have found that p21, the target of p53, is one of the major CKIs, which directly inhibit the activity of CDKs, thereby leading to cell cycle arrest in the G1 phase [49]. The exact mechanism of apoptosis and cell cycle deregulation by galaxamide and its analogues needs a further exploration of signal transduction pathways. It is, however, tempting to speculate that cyclin kinase inhibitors, cyclin dependent kinases and their regulatory cyclins proteins operating in G1 phase of the cell cycle may be involved in the galaxamide-mediated apoptosis and cell cycle arrest. Thus, present investigation revealed that the galaxamide and its analogues exerted growth inhibitory effect on SMMC-7721 cells by arresting the cell cycle in the G0/G1 phase and inducing apoptosis.

## 4. Experimental Section

### 4.1. General

NMR spectra were recorded on a Bruker Avance 300 spectrometer (300 MHz for ^1^H and 75 MHz for ^13^C) in CDCl_3_. Chemical shifts are reported as δ values in parts per million (ppm) relative to tetramethylsilane (TMS) and *J* values are expressed in Hertz. The ESI mass spectra were obtained on a LCQ DECA XP LC-MS mass spectrometer. Silica gel (200–300 mesh) for column chromatography and silica GF254 for TLC were produced by Qingdao Marine Chemical Company (Qingdao, China). All air- or moisture-sensitive reactions were conducted under nitrogen atmosphere. Starting materials and reagents used in reactions were obtained commercially from Acros, Aldrich, GL Biochem and were used without purification, unless otherwise indicated.

### 4.2. Procedure for d/l Fmoc-N-Me Leucine

#### 4.2.1. Step-1 Synthesis of Oxazolidinones from Fmoc Leu

The Fmoc Leu (5 mmol), paraformaldehyde (6.66 eq., 1 g) and *p*-toluenesulphonic acid (0.12 eq., 100 mg) were suspended in toluene (100 mL) in 250 mL three neck RBF. The mixture was refluxed for 30 min in a Dean-Stark setup and reaction was monitored by TLC (97.5:2:0.5 CHCl_3_:MeOH:AcOH). The solution was cooled, washed with saturated NaHCO_3_ and dried over anhydrous NaSO_4_. Concentration in vacuo gave the crude product which in most cases solidified upon standing. In some cases, purification via flash chromatography was required (silica gel, 3:7 ethylacetate:pentane).

#### 4.2.2. Step-2 Synthesis of *N*-methylated Fmoc Leu from Oxazolidinones

To a solution of the oxazolidinone (1 eq.) and Lewis acid (2 eq.) in dry DCM (20 mL/1 mmol oxazolidinone) was added triethylsilane (2 eq.). The reaction was stirred at ambient temperature until TLC (1:3 ethylacetate:hexane) showed the absence of starting material. An additional amount of DCM was added (twice the reaction volume) and the organic phase was washed with 1 M HCl. The organic phase was dried over anhydrous sodium sulphate and concentrated in vacuo. The crude product was purified via column chromatography on silica gel (hexane and ethylacetate).

### 4.3. General Procedure for the Synthesis of Pentapeptides-COOH

Synthesis of linear peptide was carried out using 2-chlorotrityl chloride (CTC) resin (1.05 mmol/g) following the standard Fmoc strategy. All reactions were performed on 0.250 g of resin scale. First *N*-Fmoc protected aa (2 eq.) residue was coupled with CTC resin (0.250 g) using DIPEA (4 eq.) in DCM (5 mL) for 2 h at rt. After filtration, the remaining unreacted sites were capped by a solution of DCM/MeOH/DIPEA (4:5:1, 10 mL) for 30 min. Resin was washed with DCM (3 × 5 mL), *N*,*N*-Dimethylformamide (DMF) (3 × 5 mL) MeOH (3 × 5 mL). Deprotection of Fmoc group was carried out using 20% piperidine in DMF for (2 × 10 min) at rt. Loading of resin was measured by concentration of Fmoc group by ultraviolet standard curve method. The first aa bound resin was thoroughly washed with DCM (3 × 5 mL), DMF (3 × 5 mL) and MeOH (3 × 5 mL). All other *N*-Fmoc aa (2.0 eq.) coupling, including *N*-Me were carried out using standard solid phase peptide synthesis using DEPBT (2.0 eq.) as coupling reagent in the presence of DIPEA (2 eq.) in DMF/DCM (1:1, 5 mL) at rt for 3 h. Reaction was monitored using Kaiser test for the primary amine and chloranil test for the secondary amine. Generally, coupling with *N*-Me aa took 5–6 h further time to complete the reaction. Cleavage was performed using 1% TFA in DCM for (2 × 30 min) at rt. After cleavage, filtrate was immediately neutralized with 1% pyridine in MeOH and concentrated under reduced pressure. Crude peptide was isolated via precipitation from diethylether (20 mL), collected through centrifuge as a white solid, which can be directly used for macrocyclisation reaction without further purification.

### 4.4. General Procedure for Macrocyclisation Reaction

All the macrocyclisation reaction was performed at 80 mg scale. The linear pentapeptide (80 mg, 0.126 mmol) was dissolved in dry DCM (0.0007 M, 181 mL) in 250 mL round bottom flask under nitrogen atmosphere. Coupling reagent PyBOP (2 eq., 0.253 mmol, 131 mg) and DIPEA (2 eq., 0.253 mmol, 44 μL) was added at rt. Stirred the reaction for one day and monitored the progress of reaction on TLC as well as on reverse phase HPLC using a gradient of acetonitrile and DI water with 0.1% TFA. Upon completion of reaction, work up with saturated sodium bicarbonate solution (16 mL) and ammonium chloride (16 mL) solution was carried out. Organic layer collected, dried over anhydrous sodium sulphate and concentrated under reduced pressure. Crude cyclic product was purified on preparative reverse phase column chromatography using MeOH and DI water gradient at 210 nM.

### 4.5. Spectral Data

*l Fmoc-N-Me Leucine*. Yield: 88%; ^1^H NMR (300 MHz, Chloroform-*d*) δ ppm: 12.74 (s, 1 H), 7.85 (t, *J* = 7.4 Hz, 2 H), 7.60 (td, *J* = 7.6, 3.6 Hz, 2 H), 7.43–7.23 (m, 4 H), 4.64–4.17 (m, 4 H), 2.68 (s, 3 H), 1.74–1.19 (m, 3 H), 0.89–0.63 (m, 6 H); ^13^C NMR (75 MHz, Chloroform-d) δ ppm:178.31, 156.24, 143.70(2C), 141.35(2C), 127.76(2C), 127.11(2C), 125.10(2C), 120.03(2C), 67.12, 52.42, 47.21, 41.44, 24.81, 22.91, 21.73(2C); MS (ESI) *m*/*z*: 368.4 [M + H]^+^, 385.5 [M + NH_4_]^+^, 390.6 [M + Na]^+^.

*d Fmoc-N-Me Leucine*. Yield: 89%; ^1^H NMR (300 MHz, Chloroform-*d*) δ ppm: 12.71 (s, 1 H), 7.83 (t, *J* = 7.4 Hz, 2 H), 7.61 (td, *J* = 7.6, 3.6 Hz, 2 H), 7.42–7.22 (m, 4 H), 4.63–4.16 (m, 4 H), 2.67 (s, 3 H), 1.75–1.20 (m, 3 H), 0.90–0.64 (m, 6 H); ^13^C NMR (75 MHz, Chloroform-*d*) δ ppm:178.31, 156.24, 143.70(2C), 141.35(2C), 127.76(2C), 127.11(2C), 125.10(2C), 120.03(2C), 67.12, 52.42, 47.21, 41.44, 24.81, 22.91, 21.73(2C); MS (ESI) *m*/*z*: 368.5 [M + H]^+^, 385.5 [M + NH_4_]^+^, 390.6 [M + Na]^+^.

*Compound* **1** *cyclo(Phe-d-Leu-N-Me-Leu-d-Leu-d-Leu)*. Yield: 57.3%, Wt: 44.5 mg, white powder; ^1^H NMR (300 MHz, Chloroform-*d*) δ ppm: 7.90 (dd, *J* = 24.9, 8.1 Hz, 2 H), 7.76–7.67 (m, 1 H), 7.24 (d, *J* = 13.6 Hz, 5 H), 6.94 (d, *J* = 6.9 Hz, 1 H), 5.04 (d, *J* = 8.7 Hz, 1 H), 4.79 (d, *J* = 7.7 Hz, 1 H), 4.56 (q, *J* = 10.1, 9.6 Hz, 2 H), 3.71 (dq, *J* = 14.2, 7.6, 7.2 Hz, 2 H), 3.22 (s, 3 H), 3.13 (s, 1 H), 1.58 (d, *J* = 18.4 Hz, 4 H), 1.43 (d, *J* = 8.0 Hz, 8 H), 1.06-0.71 (m, 24 H); ^13^C NMR (75 MHz, Chloroform-*d*) δ ppm: 174.67, 173.89, 173.14, 172.33, 170.78, 137.12, 129.39(2C), 128.31(2C), 126.55, 58.11, 56.75, 54.05, 51.75, 48.41, 41.63, 40.29, 38.40, 37.00, 36.80, 31.04, 25.16, 25.06, 25.00, 24.68, 23.15, 22.94, 22.83, 22.74, 22.53, 22.28, 21.42, 21.31; MS (ESI) *m*/*z*: 614.7 [M + H]^+^, 631.7 [M + NH_4_]^+^, 636.6 [M + Na]^+^.

*Compound* **2** *cyclo(d-Phe-Leu-N-Me-Leu-d-Leu-d-Leu)*. Yield: 61.5%, Wt: 47.8 mg, white powder; ^1^H NMR (300 MHz, Chloroform-*d*) δ 8.50 (d, *J* = 7.9 Hz, 1 H), 7.58 (d, *J* = 8.7 Hz, 1 H), 7.40–7.13 (m, 6 H), 6.96 (d, *J* = 8.5 Hz, 1 H), 5.04 (t, *J* = 7.5 Hz, 1 H), 4.85 (q, *J* = 7.7 Hz, 1 H), 4.49 (dd, *J* = 11.0, 6.6 Hz, 2 H), 3.81 (q, *J* = 8.5, 6.9 Hz, 1 H), 3.29 (dd, *J* = 14.2, 7.1 Hz, 1 H), 3.15 (dd, *J* = 14.1, 4.9 Hz, 1 H), 2.70 (s, 3 H), 1.85 (dd, *J* = 13.4, 6.6 Hz, 2 H), 1.55 (dtq, *J* = 40.8, 13.0, 6.9, 6.4 Hz, 10 H), 1.04–0.72 (m, 24 H); ^13^C NMR (75 MHz, CDCl_3_) δ ppm: 174.02, 171.76, 170.58, 170.03, 170.01, 135.98, 129.19(2C), 129.0(2C), 127.54, 55.05, 53.88(2C), 48.61(2C), 41.16, 40.58, 37.38, 37.25, 34.75, 29.36, 24.88, 24.80(3C), 23.05, 22.81, 22.75, 22.67, 22.51, 22.31, 21.94, 21.66; MS (ESI) *m*/*z*: 614.6 [M + H]^+^, 631.6 [M + NH_4_]^+^, 636.5 [M + Na]^+^.

*Compound* **3** *(cyclo(Phe-Leu-d-N-Me-Leu-d-Leu-d-Leu).* Yield: 51.7% , Wt: 40.2 mg, white powder; ^1^H NMR (300 MHz, Chloroform-*d*) δ ppm: 8.07 (d, *J* = 6.8 Hz, 1 H), 7.93 (d, *J* = 8.3 Hz, 1 H), 7.33–7.11 (m, 6 H), 6.75 (d, *J* = 9.1 Hz, 1 H), 5.02 (t, *J* = 7.7 Hz, 1 H), 4.91 (t, *J* = 7.0 Hz, 1 H), 4.49 (ddd, *J* = 11.3, 6.9, 4.3 Hz, 1 H), 4.36-4.10 (m, 1 H), 3.67 (q, *J* = 7.8 Hz, 1 H), 3.30 (dd, *J* = 14.1, 4.3 Hz, 1 H), 3.03 (d, *J* = 2.0 Hz, 3 H), 2.82 (dd, *J* = 14.1, 11.5 Hz, 1 H), 1.84–1.31 (m, 12 H), 1.00–0.67 (m, 24 H); ^13^C NMR (75 MHz, CDCl_3_) δ ppm: 173.39, 172.82, 172.20, 172.18, 171.19, 136.59, 128.86(2C), 128.74(2C), 127.07, 58.88, 56.74, 54.87, 52.07, 48.23, 41.54, 41.28, 38.36, 37.11, 36.65, 30.68, 25.57, 25.20, 25.15, 24.72, 22.86, 22.71, 22.63, 22.57(2C), 22.44, 22.24, 22.14; MS (ESI) *m*/*z*: 614.4 [M + H]^+^, 631.6 [M + NH_4_]^+^, 636.3 [M + Na]^+^.

*Compound* **4** *cyclo(d-Phe-Leu-d-N-Me-Leu-Leu-d-Leu).* Yield: 53.8%, Wt: 41.8 mg, white powder; ^1^H NMR (300 MHz, Chloroform-*d*) δ ppm: 7.30 (d, *J* = 7.5 Hz, 4 H), 7.22–7.12 (m, 3 H), 6.98 (d, *J* = 9.9 Hz, 1 H), 6.25 (d, *J* = 7.8 Hz, 1 H), 5.05 (t, *J* = 7.6 Hz, 1 H), 4.93–4.79 (m, 1 H), 4.63 (td, *J* = 8.8, 4.5 Hz, 1 H), 4.51-4.36 (m, 1 H), 3.85 (dt, *J* = 9.6, 6.1 Hz, 1 H), 3.31 (dd, *J* = 14.3, 4.5 Hz, 1 H), 3.04–2.91 (m, 1 H), 2.72 (s, 3 H), 1.85 (dt, *J* = 13.6, 6.9 Hz, 1 H), 1.73–1.40 (m, 10 H), 1.34 (ddd, *J* = 13.6, 8.8, 6.0 Hz, 1 H), 0.93 (ddd, *J* = 17.6, 11.4, 6.3 Hz, 18 H), 0.75 (d, *J* = 6.5 Hz, 3 H), 0.69 (d, *J* = 6.5 Hz, 3 H); ^13^C NMR (75 MHz, CDCl_3_) δ ppm: 174.38, 172.30, 171.71, 170.16, 169.99, 136.24, 128.93(2C), 128.89(2C), 127.28, 54.96, 53.90, 53.75, 51.05, 48.41, 41.30, 40.59, 39.08, 37.37, 34.61, 29.42, 24.92, 24.82, 24.77, 24.49, 22.85, 22.80, 22.78, 22.69, 22.54, 22.15, 22.04, 21.83; MS (ESI) *m*/*z*: 614.7 [M + H]^+^, 631.7 [M + NH_4_]^+^, 636.6 [M + Na]^+^.

*Compound* **5** *cyclo(d-Phe-d-Leu-N-Me-Leu-d-Leu-N-Me-Leu).* Yield: 53.9%, Wt: 41.9 mg, white powder; ^1^H NMR (300 MHz, Chloroform-*d*) δ ppm: 7.33–7.09 (m, 6 H), 7.04 (d, *J* = 9.6 Hz, 1 H), 5.97 (d, *J* = 9.3 Hz, 1 H), 5.11 (t, *J* = 7.7 Hz, 1 H), 4.92 (dd, *J* = 9.9, 4.6 Hz, 1 H), 4.80 (tt, *J* = 9.1, 4.1 Hz, 2 H), 4.16 (dd, *J* = 10.5, 5.4 Hz, 1 H), 3.22 (dd, *J* = 14.0, 4.9 Hz, 1 H), 3.13 (s, 3 H), 3.10–2.96 (m, 1 H), 2.70 (s, 3 H), 1.78–1.21 (m, 12 H), 1.00–0.69 (m, 24 H); ^13^C NMR (75 MHz, CDCl_3_) δ ppm: 175.88, 171.73, 171.51, 170.12, 170.06, 136.18, 129.17(2C), 128.79(2C), 127.25, 57.86, 53.74(2C), 48.71, 47.83, 41.45, 41.13, 37.70, 37.47, 34.46, 31.40, 29.47, 24.96(2C), 24.83, 24.55, 23.27, 23.11, 22.95(2C), 22.24, 21.97, 21.82, 21.65; MS (ESI) *m*/*z*: 628.8 [M + H]^+^, 645.8 [M + NH_4_]^+^, 650.9 [M + Na]^+^.

*Compound* **6** *cyclo(Phe-Leu-Leu-N-Me-Leu-d-Leu).* Yield: 64.5%, Wt: 50.1 mg, white powder; ^1^H NMR (300 MHz, Chloroform-*d*) δ 7.36-7.19 (m, 5 H), 7.15 (d, *J* = 9.2 Hz, 1 H), 7.02 (d, *J* = 9.2 Hz, 1 H), 6.72 (t, *J* = 7.4 Hz, 2 H), 5.07 (t, *J* = 7.6 Hz, 1 H), 4.89 (dt, *J* = 9.1, 6.8 Hz, 1 H), 4.37 (dddd, *J* = 20.4, 10.0, 8.7, 5.3 Hz, 3 H), 3.24 (dd, *J* = 14.0, 6.1 Hz, 1 H), 3.01–2.91 (m, 1 H), 2.89 (s, 3 H), 1.88 (ddd, *J* = 14.1, 9.5, 4.7 Hz, 1 H), 1.73 (dt, *J* = 13.7, 7.8 Hz, 2 H), 1.63–1.40 (m, 7 H), 1.38–1.24 (m, 2 H), 1.00–0.85 (m, 18 H), 0.79 (t, *J* = 6.4 Hz, 6 H); ^13^C NMR (75 MHz, CDCl_3_) δ ppm: 173.39, 172.88, 171.03, 170.55, 170.40, 135.72, 128.98(2C), 128.88(2C), 127.42, 56.74, 54.34, 51.83, 51.13, 48.10, 41.17, 40.83, 39.57, 37.12, 35.24, 30.18, 25.08, 24.72(3C), 23.26, 22.77, 22.74, 22.60, 22.39, 22.30(2C), 21.39; MS (ESI) *m*/*z*: 614.6 [M + H]^+^, 631.6 [M + NH_4_]^+^, 636.5 [M + Na]^+^.

*Compound* **7** *cyclo(Phe-Leu-d-Leu-N-Me-Leu-Leu).* Yield: 52.2%, Wt: 40.6 mg, white powder; ^1^H NMR (300 MHz, Chloroform-*d*) δ ppm: 7.85 (d, *J* = 8.2 Hz, 1 H), 7.68 (d, *J* = 7.4 Hz, 2 H), 7.30–7.16 (m, 5 H), 6.95 (d, *J* = 7.3 Hz, 1 H), 5.04–4.85 (m, 2 H), 4.31 (ddt, *J* = 32.9, 9.1, 6.4 Hz, 2 H), 4.11 (q, *J* = 7.5 Hz, 1 H), 3.41 (dd, *J* = 13.8, 9.2 Hz, 1 H), 3.19 (s, 3 H), 2.76–2.68 (m, 1 H), 1.87–1.49 (m, 9 H), 1.41 (dp, *J* = 20.1, 6.5 Hz, 3 H), 1.01–0.77 (m, 24 H); ^13^C NMR (75 MHz, Chloroform-*d*) δ ppm: 174.24, 173.83, 172.80, 171.67, 171.25, 136.83, 129.14(2C), 128.46(2C), 126.81, 59.24, 56.38, 54.26, 52.19, 48.18, 40.73, 40.37, 39.80, 36.88, 36.41, 30.99, 25.23, 25.05, 24.94, 24.86, 23.05, 22.89, 22.72, 22.61, 22.50, 22.28, 22.21, 21.48; MS (ESI) *m*/*z*: 614.7 [M + H]^+^, 631.7 [M + NH_4_]^+^, 636.6 [M + Na]^+^.

*Compound* **8** *cyclo(Phe-d-Leu-Leu-N-Me-Leu-Leu).* Yield: 44.8%, Wt: 34.8 mg, white powder; ^1^H NMR (300 MHz, Chloroform-*d*) δ ppm: 7.81 (d, *J* = 8.3 Hz, 1 H), 7.62 (dd, *J* = 11.7, 7.7 Hz, 2 H), 7.23 (h, *J* = 5.0 Hz, 5 H), 6.96 (d, *J* = 7.4 Hz, 1 H), 4.98 (dd, *J* = 10.1, 5.9 Hz, 1 H), 4.90 (d, *J* = 7.6 Hz, 1 H), 4.39-4.29 (m, 1 H), 4.22 (d, *J* = 9.2 Hz, 1 H), 4.11 (d, *J* = 7.5 Hz, 1 H), 3.42 (dd, *J* = 13.7, 9.3 Hz, 1 H), 3.30–3.20 (m, 1 H), 3.19 (s, 3 H), 1.73–1.52 (m, 9H), 1.42 (dp, *J* = 13.6, 6.7 Hz, 3 H), 1.03–0.79 (m, 24 H); ^13^C NMR (75 MHz, Chloroform-*d*) δ ppm: 174.25, 173.77, 172.93, 171.69, 171.20, 136.85, 129.12(2C), 128.48(2C), 126.82, 59.35, 56.34, 54.22, 52.16, 48.24, 40.64, 40.23, 39.71, 36.81, 36.36, 30.97, 25.23, 25.02, 24.95, 24.84, 23.06, 22.90, 22.73, 22.61, 22.51, 22.29, 22.16, 21.47; MS (ESI) *m*/*z*: 614.5 [M + H]^+^, 631.5 [M + NH_4_]^+^, 636.4 [M + Na]^+^.

*Compound* **9** *cyclo(d-Phe-Leu-Leu-N-Me-Leu-d-Leu).* Yield: 60.2%, Wt: 46.8 mg, white powder; ^1^H NMR (300 MHz, Chloroform-*d*) δ ppm: 7.34 (d, *J* = 9.2 Hz, 1 H), 7.30–7.17 (m, 5 H), 7.06 (d, *J* = 7.4 Hz, 1 H), 6.52 (s, 2 H), 5.07–4.87 (m, 1 H), 4.71 (d, *J* = 7.7 Hz, 1 H), 4.46 (qd, *J* = 10.7, 9.9, 4.4 Hz, 3 H), 3.13 (d, *J* = 8.2 Hz, 2 H), 3.09 (s, 3 H), 1.79–1.26 (m, 12 H), 1.04–0.65 (m, 24 H); ^13^C NMR (75 MHz, CDCl_3_) δ ppm: 174.81, 172.13, 171.18, 170.98, 170.67, 136.97, 129.31(2C), 128.44(2C), 126.68, 56.13, 55.93, 52.57, 50.69, 47.97, 41.87, 41.37, 37.59, 37.11, 36.78, 30.94, 25.11, 25.06, 24.85, 24.40, 23.05, 22.99, 22.91, 22.57, 22.52, 22.15, 21.80, 21.49; MS (ESI) *m*/*z*: 614.3 [M + H]^+^, 631.3 [M + NH_4_]^+^, 636.4 [M + Na]^+^.

*Compound* **10** *cyclo(d-Phe-Leu-d-Leu-N-Me-Leu-Leu).* Yield: 53.1%, Wt: 41.3 mg, white powder; ^1^H NMR (300 MHz, Chloroform-*d*) δ ppm: 7.49 (d, *J* = 8.6 Hz, 1 H), 7.33–7.13 (m, 5 H), 6.96 (dd, *J* = 8.8, 2.4 Hz, 1 H), 6.76 (t, *J* = 9.5 Hz, 1 H), 6.57 (dd, *J* = 9.7, 5.1 Hz, 1 H), 5.17 (dd, *J* = 11.8, 4.3 Hz, 1 H), 4.76 (q, *J* = 7.9 Hz, 1 H), 4.64 (q, *J* = 6.9 Hz, 1 H), 4.60–4.38 (m, 2 H), 3.21 (dd, *J* = 14.2, 7.4 Hz, 1 H), 3.14 (s, 3 H), 2.97–2.84 (m, 1 H), 2.13–1.36 (m, 12 H), 1.05–0.78 (m, 24 H); ^13^C NMR (75 MHz, CDCl_3_) δ ppm: 175.07, 174.57, 172.58, 172.02, 170.54, 137.03, 129.13(2C), 128.44(2C), 126.58, 56.79, 53.61, 51.78, 51.04, 49.14, 40.09, 40.04, 39.88, 36.58, 34.95, 30.97, 25.35, 24.91(2C), 24.77, 23.38, 22.94, 22.90, 22.56, 22.33, 22.23, 22.15, 20.84; MS (ESI) *m*/*z*: 614.6 [M + H]^+^, 631.5 [M + NH_4_]^+^, 636.6 [M + Na]^+^.

*Compound* **11** *cyclo(d-Phe-d-Leu-Leu-N-Me-Leu-d-Leu).* Yield: 50.4%, Wt: 39.2 mg, white powder; ^1^H NMR (300 MHz, Chloroform-*d*) δ ppm: 8.35–8.13 (m, 1 H), 7.48 (d, *J* = 8.8 Hz, 1 H), 7.31–7.13 (m, 5 H), 6.90 (d, *J* = 9.8 Hz, 1 H), 6.24 (d, *J* = 6.8 Hz, 1 H), 4.98 (t, *J* = 7.2 Hz, 1 H), 4.91–4.75 (m, 1 H), 4.48 (td, *J* = 9.3, 6.2 Hz, 1 H), 4.31–4.08 (m, 2 H), 3.27 (d, *J* = 10.0 Hz, 1 H), 3.18 (dd, *J* = 13.6, 7.0 Hz, 1 H), 2.60 (d, *J* = 1.5 Hz, 3 H), 1.90–1.74 (m, 2 H), 1.60–1.27 (m, 10 H), 1.00–0.79 (m, 24 H); ^13^C NMR (75 MHz, CDCl_3_) δ ppm: 174.32, 171.97, 171.24, 170.97, 170.20, 136.61, 128.83(2C), 128.76(2C), 126.98, 56.23, 53.89(2C), 51.46, 48.17, 41.41, 40.69, 40.63, 34.75, 34.62, 29.44, 24.86, 24.78(3C), 23.22, 22.77, 22.75, 22.70, 22.63, 22.29, 21.95, 21.04; MS (ESI) *m*/*z*: 614.6 [M + H]^+^, 631.6 [M + NH_4_]^+^, 636.4 [M + Na]^+^.

*Compound* **12** *cyclo(d-Phe-d-Leu-d-Leu-N-Me-Leu-Leu).* Yield: 46.1%, Wt: 35.8 mg, white powder; ^1^H NMR (300 MHz, Chloroform-*d*) δ ppm: 7.36–7.17 (m, 6 H), 7.10 (s, 1 H), 6.96 (d, *J* = 10.3 Hz, 2 H), 5.06 (dd, *J* = 10.6, 5.3 Hz, 1 H), 4.87 (q, *J* = 7.5, 7.1 Hz, 1H), 4.72 (dd, *J* = 17.3, 9.2 Hz, 1 H), 4.40 (q, *J* = 7.8 Hz, 1 H), 3.84 (q, *J* = 8.3, 7.7 Hz, 1 H), 3.22–3.14 (m, 1H), 3.03 (s, 3 H), 3.00–2.95 (m, 1 H), 1.86–1.40 (m, 12 H), 1.02–0.73 (m, 24 H); ^13^C NMR (75 MHz, CDCl_3_) δ ppm: 174.15, 172.69, 172.31, 171.75, 171.41, 136.65, 129.19(2C), 128.50(2C), 126.79, 56.98, 55.94, 55.11, 51.92, 48.67, 41.08, 39.72, 38.69, 36.65, 36.44, 30.74, 25.33, 24.84(2C), 24.74, 23.20, 23.05, 22.97, 22.89, 22.38, 22.24, 21.59, 21.27; MS (ESI) *m*/*z*: 614.5 [M + H]^+^, 631.5 [M + NH_4_]^+^, 636.6 [M + Na]^+^.

*Compound* **13** *cyclo(d-Phe-d-Leu-d-Leu-N-Me-Leu-Leu).* Yield: 50.3%, Wt: 39.1 mg, white powder; ^1^H NMR (300 MHz, Chloroform-*d*) δ ppm: 7.49 (d, *J* = 8.6 Hz, 1 H), 7.23 (q, *J* = 7.2, 6.7 Hz, 5 H), 6.96 (dd, *J* = 8.8, 2.4 Hz, 1 H), 6.76 (t, *J* = 9.5 Hz, 1 H), 6.57 (dd, *J* = 9.7, 5.1 Hz, 1 H), 5.17 (dd, *J* = 11.8, 4.3 Hz, 1 H), 4.76 (q, *J* = 7.9 Hz, 1 H), 4.64 (q, *J* = 6.9 Hz, 1 H), 4.57–4.43 (m, 2 H), 3.28–3.19 (m, 1 H), 3.14 (s, 3 H), 2.96–2.85 (m, 1 H), 1.94 (dtt, *J* = 34.9, 9.8, 5.1 Hz, 2 H), 1.76–1.46 (m, 10 H), 1.03–0.78 (m, 24 H); ^13^C NMR (75 MHz, Chloroform-*d*) δ ppm: 173.48, 172.91, 171.04, 170.58, 170.42, 135.73, 129.00(2C), 128.87(2C), 127.44, 56.90, 54.39, 51.83, 51.27, 48.20, 41.22, 40.82, 39.68, 37.04, 35.27, 30.20, 25.09, 24.76(2C), 24.72, 23.23, 22.77, 22.73, 22.59, 22.41, 22.33, 22.30, 21.42; MS (ESI) *m*/*z*: 614.4 [M + H]^+^, 631.4 [M + NH_4_]^+^, 636.6 [M + Na]^+^.

*Compound* **14** *cyclo(d-Phe-d-Leu-d-Leu-N-Me-Leu-d-Leu).* Yield: 55.5%, Wt: 43.1 mg, white powder; ^1^H NMR (300 MHz, Chloroform-*d*) δ ppm; 8.28 (q, *J* = 9.0, 8.3 Hz, 1 H), 7.48 (d, *J* = 8.7 Hz, 1 H), 7.31–7.16 (m, 5 H), 6.89 (d, *J* = 9.8 Hz, 1 H), 6.23 (d, *J* = 6.9 Hz, 1 H), 4.98 (t, *J* = 7.1 Hz, 1 H), 4.85 (q, *J* = 7.4 Hz, 1 H), 4.55–4.40 (m, 1 H), 4.32–4.08 (m, 2 H), 3.32–3.12 (m, 2H), 2.59 (s, 3 H), 1.83 (ddq, *J* = 18.3, 9.8, 5.1, 4.0 Hz, 2 H), 1.64–1.31 (m, 10 H), 1.00–0.78 (m, 24 H); ^13^C NMR (75 MHz, CDCl_3_) δ ppm: 174.33, 171.97, 171.23, 170.98, 170.18, 136.63, 128.84(2C), 128.74(2C), 126.96, 56.20, 53.87(2C), 51.45, 48.17, 41.41, 40.70, 40.63, 34.74, 34.62, 29.43, 24.85(2C), 24.78(2C), 23.22, 22.77, 22.74, 22.70, 22.62, 22.29, 21.95, 21.04; MS (ESI) *m*/*z*: 614.5 [M + H]^+^, 631.4 [M + NH_4_]^+^, 636.3 [M + Na]^+^.

*Compound* **15** *cyclo(Phe-Leu-Leu-d-N-Me-Leu-Leu).* Yield: 49.2%, Wt: 38.2 mg, white powder; ^1^H NMR (300 MHz, Chloroform-*d*) δ ppm: 7.98 (t, *J* = 7.6 Hz, 2 H), 7.77 (d, *J* = 6.2 Hz, 1 H), 7.25 (q, *J* = 8.6 Hz, 5 H), 6.83 (d, *J* = 6.6 Hz, 1 H), 5.06 (dd, *J* = 10.9, 5.2 Hz, 1 H), 4.81 (q, *J* = 7.4 Hz, 1 H), 4.59 (q, *J* = 7.6 Hz, 1 H), 4.48 (q, *J* = 7.1, 6.7 Hz, 1 H), 3.72 (dt, *J* = 11.4, 6.3 Hz, 1 H), 3.31 (d, *J* = 6.0 Hz, 1 H), 3.23 (s, 3 H), 3.05 (dd, *J* = 13.1, 9.5 Hz, 1 H), 2.13 (td, *J* = 12.3, 10.4, 4.9 Hz, 1 H), 2.00–1.85 (m, 1 H), 1.78–1.32 (m, 10 H), 1.06–0.81 (m, 21 H), 0.68 (d, *J* = 6.5 Hz, 3 H); ^13^C NMR (75 MHz, CDCl_3_) δ ppm: 174.28, 172.06, 171.23, 170.90, 170.23, 136.56, 128.82(2C), 128.78(2C), 127.02, 56.44, 53.98, 51.46, 50.86, 48.15, 41.39, 40.66, 40.59, 34.64, 34.78, 29.49, 24.87, 24.80, 24.78(2C), 23.22, 22.77(2C), 22.69, 22.63, 22.29, 21.95, 21.06; MS (ESI) *m*/*z*: 614.5 [M + H]^+^, 631.5 [M + NH_4_]^+^, 636.4 [M + Na]^+^.

*Compound* **16** *cyclo(Phe-d-Leu-Leu-d-N-Me-Leu-Leu).* Yield: 53.3%, Wt: 41.4 mg, white powder; ^1^H NMR (300 MHz, Chloroform-*d*) δ ppm: 7.32 (d, *J* = 9.4 Hz, 1 H), 7.26–7.20 (m, 5 H), 7.11 (d, *J* = 7.2 Hz, 1 H), 6.66–6.53 (m, 2 H), 4.96 (td, *J* = 8.7, 5.7 Hz, 1 H), 4.71 (t, *J* = 7.8 Hz, 1 H), 4.45 (dtt, *J* = 13.7, 9.3, 4.5 Hz, 3 H), 3.20–3.10 (m, 2 H), 3.08 (s, 3 H), 1.77–1.30 (m, 12 H), 1.02–0.89 (m, 18 H), 0.82 (d, *J* = 6.5 Hz, 3 H), 0.77 (d, *J* = 6.4 Hz, 3 H); ^13^C NMR (75 MHz, CDCl_3_) δ ppm: 174.74, 172.19, 171.22, 171.01, 170.72, 136.96, 129.33(2C), 128.42(2C), 126.67, 55.95, 55.76, 52.63, 50.72, 47.93, 41.79, 41.33, 37.60, 37.10, 36.77, 30.93, 25.10, 25.05, 24.84, 24.42, 23.02, 22.97, 22.90, 22.58, 22.49, 22.18, 21.82, 21.50; MS (ESI) *m*/*z*: 614.6 [M + H]^+^, 631.6 [M + NH_4_]^+^, 636.5 [M + Na]^+^.

*Compound* **17** *cyclo(d-Phe-Leu-Leu-d-N-Me-Leu-Leu).* Yield: 56.4%, Wt: 43.8 mg, white powder; ^1^H NMR (300 MHz, Chloroform-*d*) δ ppm: 8.02 (d, *J* = 7.8 Hz, 1 H), 7.33–7.14 (m, 6 H), 7.08 (dd, *J* = 14.4, 9.8 Hz, 2 H), 5.04 (t, *J* = 7.6 Hz, 1 H), 4.83 (dt, *J* = 9.3, 6.8 Hz, 1 H), 4.61–4.46 (m, 1 H), 4.26 (dt, *J* = 10.5, 6.2 Hz, 1 H), 4.06 (ddd, *J* = 11.1, 7.1, 3.6 Hz, 1 H), 3.18–3.12 (m, 1 H), 3.02 (d, *J* = 6.0 Hz, 1 H), 2.75 (s, 3 H), 1.90–1.20 (m, 12 H), 0.90 (dt, *J* = 11.6, 6.4 Hz, 18 H), 0.74 (d, *J* = 6.5 Hz, 3 H), 0.64 (d, *J* = 6.2 Hz, 3 H); ^13^C NMR (75 MHz, CDCl_3_) δ ppm: 173.77, 172.03, 171.55, 171.21, 170.21, 135.91, 129.03(2C), 128.69(2C), 127.04, 56.94, 53.95, 53.19, 51.00, 47.90, 41.47, 40.53, 40.24, 36.97, 34.87, 29.53, 24.93, 24.82, 24.69, 24.32, 23.10, 22.81, 22.74, 22.73, 22.61, 22.20, 22.10, 21.08; MS (ESI) *m*/*z*: 614.8 [M + H]^+^, 631.7 [M + NH_4_]^+^, 636.6 [M + Na]^+^.

*Compound* **18** *cyclo(d-Phe-d-Leu-Leu-d-N-Me-Leu-Leu).* Yield: 56.2%, Wt: 43.7 mg, white powder; ^1^H NMR (300 MHz, Chloroform-*d*) δ ppm: 7.49 (d, *J* = 8.9 Hz, 1 H), 7.24 (q, *J* = 6.9 Hz, 6 H), 7.12 (d, *J* = 7.0 Hz, 1 H), 6.81–6.65 (m, 1 H), 4.96 (t, *J* = 8.0 Hz, 1 H), 4.81 (t, *J* = 8.0 Hz, 2 H), 4.51 (td, *J* = 9.3, 4.1 Hz, 1 H), 4.26 (q, *J* = 7.5 Hz, 1 H), 3.23 (dd, *J* = 14.3, 6.2 Hz, 1 H), 3.06 (s, 3 H), 2.95 (dd, *J* = 14.3, 9.1 Hz, 1 H), 1.74–1.34 (m, 12 H), 1.01–0.73 (m, 24 H); ^13^C NMR (75 MHz, CDCl_3_) δ ppm: 173.50, 173.47, 172.91, 171.04, 170.42, 135.72, 129.00(2C), 128.87(2C), 127.44, 56.93, 54.38, 51.81, 51.30, 48.21, 41.22, 40.80, 39.68, 37.02, 35.25, 30.20, 25.08, 24.75(2C), 24.72, 23.24, 22.78, 22.73, 22.59, 22.41, 22.33, 22.29, 21.41; MS (ESI) *m*/*z*: 614.6 [M + H]^+^, 631.6 [M + NH_4_]^+^, 636.5 [M + Na]^+^.

*Compound* **19** *cyclo(d-Phe-d-N-Me-Leu-Leu-N-Me-Leu-Leu).* Yield: 45.5%, Wt: 35.4 mg, white powder; ^1^H NMR (300 MHz, Chloroform-*d*) δ ppm: 7.28–7.14 (m, 5 H), 6.88 (d, *J* = 8.6 Hz, 1 H), 6.82 (d, *J* = 7.3 Hz, 1 H), 6.54 (d, *J* = 9.6 Hz, 1 H), 5.30–5.02 (m, 2 H), 4.67 (q, *J* = 7.8, 7.3 Hz, 1 H), 4.45 (dt, *J* = 8.0, 3.8 Hz, 1 H), 3.43 (dd, *J* = 8.9, 4.5 Hz, 1 H), 3.32 (dt, *J* = 10.7, 5.3 Hz, 1 H), 3.08 (s, 3H), 2.93 (s, 3 H), 2.88 (d, *J* = 6.4 Hz, 1 H), 1.74–1.38 (m, 12 H), 0.99-0.74 (m, 24 H); ^13^C NMR (75 MHz, CDCl_3_) δ ppm: 173.91, 173.12, 172.52, 171.48, 170.89, 136.81, 129.32(2C), 128.36(2C), 126.69, 66.77, 56.19, 51.69, 51.28, 48.48, 40.36, 40.19, 40.02, 38.14, 37.46, 36.86, 30.86, 25.30, 25.15, 24.87, 24.83, 23.40, 23.31, 23.00, 22.90, 22.22, 22.12, 21.47, 21.17; MS (ESI) *m*/*z*: 628.7 [M + H]^+^, 645.9 [M + NH_4_]^+^, 650.9 [M + Na]^+^.

*Compound* **20** *cyclo(Phe-Leu-Leu-d-Leu-N-Me-Leu).* Yield: 46.1%, Wt: 35.8 mg, white powder; ^1^H NMR (300 MHz, Chloroform-*d*) δ ppm: 7.95 (t, *J* = 7.9 Hz, 2 H), 7.73 (d, *J* = 6.3 Hz, 1 H), 7.33–7.17 (m, 5 H), 6.88 (d, *J* = 6.8 Hz, 1 H), 5.05 (dd, *J* = 10.8, 5.2 Hz, 1 H), 4.80 (d, *J* = 7.3 Hz, 1 H), 4.59 (s, 1 H), 3.71 (s, 1 H), 3.35–3.25 (m, 1 H), 3.23 (s, 3 H), 3.08 (dd, *J* = 13.2, 9.2 Hz, 1 H), 2.12 (ddd, *J* = 13.7, 10.2, 5.2 Hz, 1 H), 1.97–1.84 (m, 1 H), 1.58 (dt, *J* = 15.4, 4.4 Hz, 7 H), 1.21–1.05 (m, 3 H), 1.08–0.66 (m, 24 H); ^13^C NMR (75 MHz, Chloroform-*d*) δ ppm: 174.72, 173.98, 173.17, 172.32, 170.71, 137.12, 129.43(2C), 128.28(2C), 126.52, 58.33, 56.93, 56.41, 51.84, 48.37, 41.77, 40.25, 38.27, 37.05, 36.83, 31.06, 25.18, 25.09, 25.02, 24.66, 23.17, 22.91, 22.84, 22.74, 22.54, 22.37, 21.39, 21.30; MS (ESI) *m*/*z*: 614.7 [M + H]^+^, 631.7 [M + NH_4_]^+^, 636.6 [M + Na]^+^.

*Compound* **21** *cyclo(Phe-d-Leu-d-Leu-d-Leu-N-Me-Leu).* Yield: 55.1%, Wt: 42.8 mg, white powder; ^1^H NMR (300 MHz, Chloroform-*d*) δ ppm: 7.43 (d, *J* = 8.8 Hz, 1 H), 7.30–7.19 (m, 6 H), 7.06 (s, 1 H), 6.89 (d, *J* = 8.5 Hz, 1 H), 4.90–4.79 (m, 1 H), 4.71 (t, *J* = 7.7 Hz, 1 H), 4.35 (d, *J* = 7.7 Hz, 2 H), 4.11 (d, *J* = 9.8 Hz, 1 H), 3.32–3.18 (m, 2 H), 3.06 (s, 3 H), 1.65–1.43 (m, 12 H), 1.00–0.86 (m, 24 H); ^13^C NMR (75 MHz, CDCl_3_) δ ppm: 174.50, 172.67, 172.53, 171.60, 171.40, 137.01, 129.40(2C), 128.36(2C), 126.71, 57.63, 55.68, 54.29, 52.91, 48.48, 41.39, 39.36, 39.17, 37.22, 36.89, 30.94, 25.26, 25.24, 24.87, 24.77, 23.02, 22.97, 22.94, 22.80, 22.44, 21.99, 21.85, 21.31; MS (ESI) *m*/*z*: 614.7 [M + H]^+^, 631.6 [M + NH_4_]^+^, 636.6 [M + Na]^+^.

*Compound* **22** *cyclo(d-Phe-Leu-Leu-d-Leu-N-Me-Leu).* Yield: 65.4%, Wt: 50.8 mg, white powder; ^1^H NMR (300 MHz, Chloroform-*d*) δ ppm: 7.65–7.45 (m, 1 H), 7.40 (d, *J* = 8.7 Hz, 1 H), 7.26 (dt, *J* = 15.9, 6.0 Hz, 5H), 6.96–6.81 (m, 1 H), 6.74 (s, 1 H), 5.18 (t, *J* = 7.6 Hz, 1 H), 4.81 (q, *J* = 7.4 Hz, 1 H), 4.71 (d, *J* = 7.3 Hz, 1 H), 4.51 (td, *J* = 9.2, 5.2 Hz, 1 H), 3.88 (dt, *J* = 9.9, 5.0 Hz, 1 H), 3.13 (dd, *J* = 13.3, 5.8 Hz, 1 H), 2.99 (s, 1 H), 2.93 (s, 3 H), 1.89–1.12 (m, 12 H), 1.06–0.68 (m, 24 H); ^13^C NMR (75 MHz, CDCl_3_) δ ppm: 173.38, 172.16, 172.01, 171.98, 170.05, 136.36, 129.22(2C), 128.68(2C), 127.03, 55.08, 54.31, 54.14, 50.99, 48.26, 40.57, 40.43, 40.05, 39.80, 35.11, 30.12, 24.98, 24.97, 24.74, 24.48, 23.17, 22.87, 22.80(2C), 22.50, 22.35, 21.70, 21.57; MS (ESI) *m*/*z*: 614.6 [M + H]^+^, 631.6 [M + NH_4_]^+^, 636.5 [M + Na]^+^.

*Compound* **23** *cyclo(d-Phe-Leu-d-Leu-Leu-N-Me-Leu).* Yield: 48.5%, Wt: 37.7 mg, white powder; ^1^H NMR (300 MHz, Chloroform-*d*) δ ppm: 7.98 (t, *J* = 7.6 Hz, 1 H), 7.77 (d, *J* = 6.2 Hz, 1 H), 7.25 (q, *J* = 8.5 Hz, 6 H), 6.83 (d, *J* = 6.6 Hz, 1 H), 5.06 (dd, *J* = 10.9, 5.2 Hz, 1 H), 4.81 (q, *J* = 7.4 Hz, 1H), 4.60 (t, *J* = 7.8 Hz, 1 H), 4.48 (q, *J* = 7.1, 6.7 Hz, 1 H), 3.72 (dt, *J* = 11.4, 6.3 Hz, 1 H), 3.31 (d, *J* = 6.0 Hz, 1 H), 3.27 (s, 3H), 3.05 (dd, *J* = 13.1, 9.5 Hz, 1 H), 2.21–1.75 (m, 3 H), 1.73–1.36 (m, 9 H), 1.01–0.63 (m, 24 H); ^13^C NMR (75 MHz, CDCl_3_) δ ppm: 174.59, 173.88, 173.14, 172.31, 170.65, 137.14, 129.43(2C), 128.26(2C), 126.49, 58.39, 57.10, 56.35, 51.83, 48.26, 41.98, 40.31, 38.23, 37.11, 36.87, 31.02, 25.18, 25.09, 25.03, 24.60, 23.19, 22.93, 22.87, 22.73, 22.55, 22.40, 21.34(2C), ; MS (ESI) *m*/*z*: 614.7 [M + H]^+^, 631.7 [M + NH_4_]^+^, 636.6 [M + Na]^+^.

*Compound* **24** *cyclo(Phe-Leu-Leu-Leu-d-N-Me-Leu).* Yield: 66.4%, Wt: 51.6 mg, white powder; ^1^H NMR (300 MHz, Chloroform-*d*) δ ppm: 8.55 (d, *J* = 7.2 Hz, 1 H), 7.54 (d, *J* = 8.9 Hz, 1 H), 7.35–7.12 (m, 7 H), 5.07 (t, *J* = 7.6 Hz, 1 H), 4.85 (td, *J* = 11.7, 10.3, 7.1 Hz, 2 H), 4.17 (p, *J* = 5.5, 4.6 Hz, 1 H), 3.68 (td, *J* = 9.5, 8.0, 4.2 Hz, 1 H), 2.98 (qd, *J* = 13.7, 8.0 Hz, 2 H), 2.72 (s, 3 H), 1.84 (tt, *J* = 11.5, 5.8 Hz, 3 H), 1.53 (dtd, *J* = 35.9, 13.2, 5.7 Hz, 9 H), 0.97–0.71 (m, 24 H); ^13^C NMR (75 MHz, CDCl_3_) δ ppm: 172.73, 172.14, 171.84, 171.24, 170.36, 136.19, 129.17(2C), 128.44(2C), 126.80, 53.96, 53.94, 53.86, 53.69, 48.13, 41.37, 40.92, 38.15, 37.26, 34.65, 29.39, 25.18, 24.75, 24.68, 24.21, 23.58, 23.13, 22.84, 22.73, 22.66, 22.19, 21.34, 20.96; MS (ESI) *m*/*z*: 614.6 [M + H]^+^, 631.6 [M + NH_4_]^+^, 636.6 [M + Na]^+^.

*Compound* **25** *cyclo(d-Phe-Leu-d-Leu-d-Leu-d-N-Me-Leu).* Yield: 50.0%, Wt: 38.8 mg, white powder; ^1^H NMR (300 MHz, Chloroform-*d*) δ ppm: 7.60 (d, *J* = 6.2 Hz, 1 H), 7.32–7.18 (m, 6 H), 7.06 (d, *J* = 8.7 Hz, 1 H), 6.52 (d, *J* = 9.4 Hz, 1 H), 6.32 (d, *J* = 9.1 Hz, 1H), 5.08 (dd, *J* = 11.8, 4.3 Hz, 1 H), 4.85 (q, *J* = 7.9 Hz, 1 H), 4.55 (p, *J* = 8.5, 7.5 Hz, 3 H), 3.40 (dd, *J* = 13.8, 7.1 Hz, 1 H), 3.02 (s, 3 H), 2.97 (d, *J* = 7.9 Hz, 1 H), 1.94–1.26 (m, 12 H), 0.91 (ddt, *J* = 29.8, 10.5, 5.4 Hz, 24 H); ^13^C NMR (75 MHz, CDCl_3_) δ ppm: 175.43, 174.81, 172.61, 171.77, 170.37, 137.61, 129.59(2C), 128.18(2C), 126.46, 56.65, 53.77, 51.76, 50.67, 49.26, 40.43, 39.64, 37.24, 37.06, 36.29, 31.04, 25.22, 24.88(2C), 24.55, 23.33, 22.86, 22.76, 22.69, 22.33, 22.20, 22.09, 20.75; MS (ESI) *m*/*z*: 614.6 [M + H]^+^, 631.6 [M + NH_4_]^+^, 636.5 [M + Na]^+^.

*Compound* **26** *cyclo(d-Phe-N-Me-Leu-Leu-Leu-d-N-Me-Leu)* Yield: 45.1%, Wt: 35.1 mg, white powder; ^1^H NMR (300 MHz, Chloroform-*d*) δ ppm: 7.29–7.17 (m, 6 H), 6.88 (d, *J* = 8.4 Hz, 1 H), 6.66 (d, *J* = 9.5 Hz, 1 H), 5.30 (dd, *J* = 11.5, 4.4 Hz, 1 H), 5.13 (td, *J* = 10.3, 5.0 Hz, 1 H), 4.57 (dq, *J* = 22.5, 7.4, 7.0 Hz, 2 H), 3.35 (dd, *J* = 10.9, 4.5 Hz, 1 H), 3.09 (s, 3 H), 3.05–3.00 (m, 1 H), 2.98 (s, 3 H), 2.91–2.87 (m, 1 H), 2.33–2.19 (m, 1 H), 2.07–1.86 (m, 2 H), 1.79–1.37 (m, 9 H), 0.99–0.78 (m, 24 H); ^13^C NMR (75 MHz, CDCl_3_) δ ppm: 173.84, 172.88, 171.50, 170.82, 170.31, 137.09, 129.69(2C), 128.29(2C), 126.67, 56.15, 55.53, 55.12, 51.89, 48.53, 40.24, 37.86, 37.77, 36.17, 35.43, 30.92, 25.18, 25.10, 24.97, 24.85, 23.38, 23.02, 22.79, 22.75, 22.62, 22.13, 21.58, 20.81; MS (ESI) *m*/*z*: 628.7 [M + H]^+^, 645.8 [M + NH_4_]^+^, 650.7 [M + Na]^+^.

### 4.6. In Vitro Anti-Tumor Activity

#### 4.6.1. Maintenance of Cell Culture

Lung cancer A549, drug-resistant lung cancer A549-DPP, human hepatocellular carcinoma HepG2 and hepatocellular carcinoma SMMC-7721 cells obtained from the China cell bank of the Institute of Biochemistry and were cultured in DMEM culture medium (DMEM, Corning, NY, USA) containing 10% fetal bovine serum (FBS, Hyclone, Logan, UT, USA), 1% penicillin-streptomycin and an antifungal agent in a 5% CO_2_ humidified atmosphere at 37 °C. The culture medium was replaced once in a day. Trypsin digestion method was adopted for cell propagation. Upon reaching 80%–90% confluence, the cells were rinsed twice with PBS. A certain amount of 0.25% trypsin digestion solution was then added and maintained for 3–5 min at 37 °C. Afterward, DMEM culture medium containing 10% fetal bovine serum was added to terminate the digestion. The cells were then blown well to form single cell suspensions.

#### 4.6.2. MTT Assay

The cell viability was determined by measuring the ability of cells to transform MTT (Genview, Houston, TX, USA) to a purple formazan dye. The cells were seeded in 96-well tissue culture plates at 2.5 × 10^3^ cells/well for 24 h. The cells were then incubated with galaxamide and its analogues for 48 h. After incubation, 20 μL/well of MTT solution (5 mg/mL phosphate buffered saline) were added and incubated for 5 h. The medium was aspirated and replaced with 100 μL/well of DMSO to dissolve the formazan salt. The color intensity of the formazan solution, which reflects the cell growth condition, was measured at 570 nm using a microplate spectrophotometer (SpectroAma™ 250, Winooski, VT, USA).

#### 4.6.3. Cell Apoptotic Analysis

The apoptotic cells were quantified using an Annexin V-FITC (Sigma-Aldrich, St. Louis, MO, USA) cell apoptosis assay kit according to instruction provided with kit. In brief, about 1.5 × 10^5^ cells were plated in 6-well plates and treated with galaxamide and representative analogues (4, 8, 16 μg/mL) for 48 h. The cells were resuspended in 200 mL binding buffer. Afterward, 5 mL annexin V-fluorescein isothiocyanate (FITC) was added and then incubated in darkness at room temperature for 10 min. The cells were again resuspended in 200 mL binding buffer and stained with 5 mL PI. The prepared cells were then analyzed using a flow cytometry (Coulter Epics Elite, Miami, FL, USA). The cells in the FITC-positive and PI-negative fraction were regarded as apoptotic cells.

#### 4.6.4. Cell Morphological Observation

Exponentially growing SMMC-7721 cells (1 × 10^5^ cells/mL) were incubated for 48 h with 4, 8, 16 μg/mL of galaxamide and representative analogues. Apoptotic nuclear morphology was visualized by the Hoechsst33342 staining technique. Cells were fixed with 3.7% of paraformaldehyde for 10 min at room temperature and washed three times with PBS. After fixation, cells were stained using Hoechsst33342 (10 μg/mL) and incubated in the darkness for 10 min. After washing three times with PBS, cells were visualized using fluorescence microscope.

#### 4.6.5. Cell Cycle Analysis

SMMC-7721 cells with the density of about 1.5 × 10^5^ were incubated with galaxamide and representative analogues (4, 8, 16 μg/mL) for 48 h. Afterwards, collected cells were washed twice with PBS and centrifugation (1000 rpm, 5 min), then fixed using 70% ethanol for 12 h at 4 °C. Ethanol was removed by centrifugation (2000 rpm, 5 min), and the cells were washed twice with PBS. Cells were then resuspended in 200 mL PI and kept at 37 °C for 15 min. The cell cycle was analyzed by flow cytometry measuring the amount of PI-labeled DNA in fixed cells.

### 4.7. Statistical Analysis

Experimental data were expressed as the mean ± SD from three independent experiments. The experimental results were analyzed statistically using SPSS software. The statistically significant differences were analyzed using Tukey’s test. *p* values < 0.05 were considered statistically significant, and *p* < 0.01 considered extremely significant.

## 5. Conclusions

In conclusion, we have demonstrated robust parallel solid phase strategy for the synthesis of galaxamide analogues, and synthesized 26 novel compounds with change of l aminoacid to d enantiomeric aminoacid, changing the position of *N*-Me aminoacid residue and substitute one Leu to d/l Phe aminoacid residue with respect to parent structure. All compounds were evaluated in vitro anti-tumor activity using MTT assay. The thorough SAR exploration provides insight of structural motif and cytotoxicity correlation, and importance of *N*-Me at 3 position with this template compound. In particular, Compound **1** exhibited promising activity to be a potential lead compound against SMMC-7721 cancer cell line which was 9 and 10 fold higher than galaxamide and current cancer drug DPP, respectively. Galaxamide analogues arrest the cell cycle in the G0/G1 phase and induce apoptosis, which confirm with representative Compound **6**, **8** and **22**. Synthesis of next generation compound utilizing information described here will be of great help to understand the clear SAR and find future lead for the cancer.
